# How does social evaluation influence Hot and Cool inhibitory control in adolescence?

**DOI:** 10.1371/journal.pone.0257753

**Published:** 2021-09-30

**Authors:** Lison Bouhours, Anaëlle Camarda, Monique Ernst, Anaïs Osmont, Grégoire Borst, Mathieu Cassotti

**Affiliations:** 1 Université de Paris, LaPsyDÉ, CNRS, Paris, France; 2 Center for Management Science, Tech-PSL Research University, MINES Paris, Paris, France; 3 National Institute of Mental Health, Bethesda, Maryland, United States of America; 4 PSYCLE (EA3273), Aix Marseille University, Aix-en-Provence, France; 5 Institut Universitaire de France, Paris, France; Institut VEDECOM, FRANCE

## Abstract

The aim of the present study is to examine whether in Hot, i.e., affectively charged contexts, or cool, i.e., affectively neutral contexts, inhibitory control capacity increases or decreases under social evaluation in adolescents and adults. In two experiments, adolescents and young adults completed two *Stroop*-like tasks under either a social evaluation condition or an alone condition. The social evaluation condition comprised the presence of a peer (Experiment 1) or an expert (Experiment 2) playing the role of an evaluator, while under the alone condition, the task was performed alone. In the *Cool Stroop* task, participants had to refrain from reading color names to identify the ink color in which the words were printed. In the *Hot Stroop* task, participants had to determine the emotional expression conveyed by faces from the *NimStim* database while ignoring the emotion word displayed beneath. The results were similar in both experiments. In adolescents, social evaluation by a peer (Experiment 1) or by an expert (Experience 2) facilitated Hot but not cool inhibitory control. In adults, social evaluation had no effect on Hot or cool inhibitory control. The present findings expand our understanding of the favorable influence of socioemotional context on Hot inhibitory control during adolescence in healthy individuals.

## Introduction

The ability to inhibit automatisms or inappropriate actions to adapt to conflicting situations is a fundamental process of cognitive and socioemotional development [1, 2]. Indeed, developmental studies converge in showing that inhibitory control is a core process involved in many domains of cognition, including arithmetic [3], reasoning and decision making [1, 2, 4], and creative thinking [5]. Although decades of social psychology research have shown that social evaluation can positively influence adults’ ability to inhibit irrelevant but prepotent responses or strategies [6–10], less is known about the direct influence of social pressure on adolescents’ inhibitory control performance [11, 12]. This lack of knowledge is surprising given that recent investigations provide evidence that adolescence is a specific time-window during which the effect of social context appears to be particularly important [13–17] and inhibitory control in response to affectively charged conflicts is more difficult [18–20]. Therefore, the aim of the present study is to examine whether Hot (i.e., affectively charged) or cool (i.e., affectively neutral) inhibitory control [18–26] varied under social evaluation among adolescents and adults.

There is growing evidence that adolescents are particularly sensitive to social presence and social evaluation, but studies on the social influence on inhibitory control during adolescence have provided incongruous results [11, 13, 27, 28]. For example, a recent study suggests that the presence of an adult may facilitate the inhibitory control of risky behaviors during adolescence [11]. Indeed, adolescents took fewer risks in the presence of their mothers compared to when they were alone, and this behavioral pattern was associated with the enhanced activity of the neural system underlying inhibitory control (i.e., the ventrolateral prefrontal cortex). Critically, even if behavioral studies have confirmed that adolescents’ risk taking increased when there are in group of same-age peers [29–31], this result disappear when one adolescent was replaced by an adult [32]. In addition, adolescents tend to prioritize the advice of older adults over that of peers in probabilistic learning task [33].

In contrast, other studies suggest that peer presence increases adolescents’ risk-taking behaviors by heightening the motivational salience of the potential immediate rewards rather than by decreasing inhibitory control capacity per se [27, 28], but see [12]. Indeed, using a computerized risky driving task, Chein et al. [27] reported that risk-taking behaviors increased in the presence of peers among adolescents but not adults [see also 29]. Moreover, neuroimaging results indicate that the risk-promoting effect of peer presence observed in adolescents is associated with enhanced activation in brain regions known to be implicated in reward sensitivity, i.e., the ventral striatum and the orbitofrontal cortex. In contrast, the prefrontal brain regions involved in executive control, including inhibitory control, are not influenced by the presence of peers in either adolescents or adults. These findings could be interpreted in terms of the dual systems model, a model that proposes that adolescent risk-taking propensity is linked to an imbalance between the relative maturity of brain structures involved in incentive processing and the relative immaturity of the brain structures involved in cognitive control [30]. Taken together, these results might suggest that different social contexts can have opposite effects on risk-taking, which may depend on the modulation of the executive control system or the incentive system. In the same vein, using a Go-NoGo task to assess the potential influence of peers’ presence on the inhibitory control performance of adolescents, a recent neuroimaging study reported a lack of effect of social context on inhibitory control ability [28]. In this study, adolescents performed the Go-NoGo task under either a social evaluation condition (i.e., being watched by an anonymous peer) or a control condition (i.e., task performed alone). The authors [28] found that the presence of a peer did not influence inhibitory control performance or the activation of brain regions involved in inhibitory control during adolescence. However, the authors noted that “it is possible that a more difficult measure of cognitive control, or one that required control over emotional or affectively evocative stimuli, would yield more substantial social context effects” [23 p. 294]. Indeed, previous developmental studies have highlighted the need to distinguish between Hot (i.e., affectively charged contexts: motivationally and emotionally relevant) and cool (i.e., affectively neutral contexts) executive functions (EFs) [22–26]. Initial support for this distinction comes from cross-sectional studies comparing the developmental progression of Hot or cool EFs in children and adolescents [22, 23, 25]. Results typically reported that both cool and Hot EFs developed linearly but cool EFs developed more rapidly than Hot EFs. Moreover, while cool and Hot EFs capabilities seems correlated in children, they are not related in adolescents suggesting that these two types of EFs become increasingly more specific with age [18, 19, 23, 25]. The functional dissociation of Hot and cool EFs that emerges during adolescent is also support by lesion studies in adult showing that impairment in typical Hot EF tasks can appear without impairments of cool EF tasks [23, 25]. Nevertheless, it should be noted that Hot and cool EFs were initially assessed by using different tasks such as the Iowa Gambling Task (IGT, assessing Hot EFs) or the Color-Word Stroop task (assessing cool EFs) which varied not only by their affective significance, but also by the cognitive processes required to solve task [18]. While the Color-Word Stroop task involved inhibitory control (i.e. in this task, participants must inhibit the color denoted by a word to identify the ink color of that word), the IGT (i.e. a task initially developed to simulate complex ambiguous decision making) required emotion based reversal learning but also working memory and inhibitory control [34]. To better characterized age related changes in Hot and cool EFs, a series of developmental studies have specifically measured inhibitory control by using affective versus neutral go/nogo [26] or Stroop tasks [18, 19]. For instance, one study reported that Hot inhibitory control abilities, assessed using affective and neutral version of the Stroop task, follow a quadratic developmental trajectory (with a trough during adolescence), whereas cool inhibitory control abilities develop linearly with age [18]. In addition, cool inhibitory control performance appear not to be related to Hot inhibitory control abilities in adolescents and adults [18]. This finding seems particularly important because Hot but not cool inhibitory control performance are linked to reducing risk-taking in adolescents [19]. Taken together these data suggest that Hot and cool inhibitory control rely on different processes and thus could be affected differently by different social contexts at different ages. In line with this hypothesis, a recent neuroimaging study have examined the combined effects of virtual peer presence, social cues, and rewards on adolescent inhibitory control using an emotional versus neutral go/nogo task [35, 36]. Results reported that virtual peer presence reduces adolescents’ inhibitory control performance in the context of social cues and anticipation of reward. In addition, theses behavioral results were related to higher orbitofrontal activations in the presence of peers relative to when the adolescents were alone. The results of this study suggest that social context might decrease inhibitory control when both sustained positive arousal and reward anticipation are manipulated. However, it does not allow to determine whether this social influence requires the presence of rewards or might occurs with stimuli that only elicit emotional arousal.

The assumption that specific social contexts impact inhibitory control in unique ways is further supported by studies showing that social presence, including the social presence of a confederate (e.g., coactor, peers) or of an experimenter [6–10], increases performance on the Color-Word Stroop task in adults. This social facilitation effect (i.e. increasing performance under social presence) is in line with the distraction/conflict theory [37], which posits that the social presence of an adult can create a distraction resulting in a cognitive overload that in turn reduces attentional focus. From this theoretical perspective, when the task is simple or requires attention on a small number of central cues, such as in the Color-Word Stroop task (i.e., the ink color), the social presence facilitates individual performance. On the other hand, if the task is complex or requires attention to a high number of cues, the social presence impairs performance [37]. These studies have demonstrated that the social facilitation effect is stronger under social evaluation (i.e. under the presence of other people with a high evaluation potential such as the experimenter). It should be noted that the social facilitation effect observed in inhibitory control tasks in adults is in sharp contrast with predictions based on another influential model of the impact of social context on cognitive performance. Indeed, to explain how social context could positively impact performance under some circumstances and negatively under others, Zajonc [38] proposed that the social context increases the salience of the more dominant or automatic response associated with a specific task. Therefore, according to Zajonc’s theory, the social context should facilitate the dominant response that the participants have to inhibit and thus impede performance in inhibitory control tasks. However, this prediction is not in line with the social facilitation effect observed in numerous studies [9, 10].

The aim of the two experiments reported in the present study was thus to determine more systematically whether social evaluation by a peer (Experiment 1) or by an expert (Experiment 2) modulates Hot and cool inhibitory control performance in the same way in adolescents and young adults.

## Experiment 1 (peer evaluator)

In Experiment 1, to determine whether social evaluation by a peer influences Hot and cool inhibitory control performance, 12 to 14-year-old adolescents (i.e., mid-puberty period, [39]) and 18 to 23-year-old young adults completed Cool and Hot Stroop tasks similar to those designed by Aïte et al. [18] in a social evaluation (i.e., in the presence of a peer evaluator) or in an alone condition (i.e., tasks performed alone). In the Cool Stroop task, participants were instructed to determine the ink color of a printed word under a condition in which the ink color was congruent with the color denoted by the word or in an incongruent condition in which the ink color was incongruent with the color denoted by the word. In the Hot Stroop task, participants were shown black-and-white pictures of faces with a word denoting an emotion printed beneath them and were asked to identify the emotion of the face. Under the congruent condition, the facial expression was congruent with the emotion denoted by the word. Under the incongruent condition, the facial expression was incongruent with the emotion denoted by the word. We reasoned that if peer presence has no effect on inhibitory control, as suggested by some previous studies [27, 28], and only influences reward sensitivity [27], then performance in the Hot and the Cool Stroop tasks should not be affected by the social evaluation of a peer in adolescents and in young adults. In contrast, if social evaluation decreases inhibitory control performance only in emotionally charged contexts and, in particular, in adolescents who are hypersensitive to such contexts [28, 35], then adolescents’ performance in the Hot but not the Cool Stroop task should be lower under peer evaluation. Last, if social evaluation increases inhibitory control [7, 9], then performance in both the Hot and the Cool Stroop tasks should increase under social evaluation in both age groups.

### Material and method

#### Participants

We recruited a sample of 101 participants (51 young adult undergrads and 50 adolescent high-schoolers; see Table 1 for demographics) randomly assigned to one of two social-context conditions: 1) an alone condition with no social context, in which the participant was alone in the experiment room (alone condition, AC) and 2) a social-evaluation condition, in which a peer was present to allegedly evaluate the participant (social-evaluation condition, SE). Adolescents were recruited from two middle schools and young adults were recruited from a university in Paris, France. Data regarding the socioeconomic background of the participants were not collected. Note that all participants attended middle schools (adolescents) and a university (young adults) serving a diverse population with a wide range of socioeconomic status.

**Table 1:** Characteristics of the sample distribution.

	Group	Condition	N	Mean Age (SD)	Puberty Score (SD)	Male (Female)
**EXPERIMENT 1**	Adolescents	AC	24	13.1 (0.5)	12.9 (3.2)	12 (12)
		SE	26	13.0 (0.5)	13.0 (4.0)	11 (15)
	Young adults	AC	24	21.3 (4.2)	19.2 (1.0)	8 (16)
		SE	27	20.3 (1.5)	19.5 (0.9)	13 (14)
**EXPERIMENT 2**	Adolescents	AC	21	12.9 (0.9)	11.9 (3.3)	12 (9)
		SE	24	13.2 (0.8)	12.6 (3.4)	15 (9)
	Young adults	AC	23	20.4 (1.0)	18.9 (1.3)	10 (13)
		SE	22	20.5 (1.4)	19.2 (0.9)	11 (11)

Gender and mean age distributions for each task condition (alone: AC; social evaluation: SE) and age group. Moreover, given that cognitive development during adolescence is strongly influence by pubertal maturation [40], we have controlled that our experimental groups (AC vs. SE) did not differ in terms of puberty score. This puberty score was obtained using a self-assessment measure of pubertal development on five categories of physical modifications (e.g., skin, growth spurt, menstruations) using a four-point Likert scale [41]. Group comparisons via ANOVAs and Chi-squared analyses revealed that the social conditions and the control groups in the adolescent and adult groups did not differ in age (Experiment 1: Adolescents: F(1, 48) < 1; Young Adults: F(1, 48) = 1.57, p = .21; Experiment 2: Adolescents: F(1, 43) = 2.1, p = .16; Young adults: F(1, 43) < 1), mean puberty score (Experiment 1: Adolescents: F(1, 48) < 1; Young Adults: F(1, 48) = 1.40, p = .24; Experiment 2: Adolescents: F(1,43) = 1.14, p = .29; Young Adults: F(1, 43) < 1) and gender (Experiment 1: Adolescents: χ^2^ = 0.3, p = .59; Young Adults: χ^2^ = 1.15, p = .28; Experiment 2: Adolescents: χ^2^ = 0.13, p = .71; Young adults: χ^2^ = 0.19, p = .66).

Sample size was determined a priori by running a priori power analysis using G*Power 3.1.9.2 [42], revealing that a minimum of 84 participants was required to detect a medium effect size of 0.25 (according to Cohen’s effect size conventions and based on previous studies reporting medium to large effect sizes of the social influence on risk taking and cognitive control, see for example [25, 37] on the Age groups (adolescents vs. young adults) Social conditions (AC vs SE) x stroop tasks (cool vs. Hot) interaction with a power (1 - β) set at.80 and α set at.05.

We obtained informed written consent from parents and all adolescents. Participants were tested in accordance with national and international norms governing the use of human research participants. The Faculty of Psychology (Comité d’éthique et de recherche) granted the ethical permission to conduct this study.

#### Stimuli

The tasks were similar to those developed by Aite et al. [18]. The *Cool Stroop* task comprised the three color words “RED,” “BLUE” and “YELLOW” printed in 24-pt Courier New font capital letters and displayed in the center of the screen on a gray background. Stimuli sustained on average 4.12° x 10.05° of visual angle. These three words were written in three different ink colors, namely, red, blue and yellow, yielding congruent trials and incongruent trials. In congruent trials, the ink color matched the meaning of the word. In incongruent trials, the ink color differed from the meaning of the word (e.g., YELLOW printed in red ink), representing a conflict requiring participants to inhibit the prepotent response of reading the word (e.g., yellow). Nine Stroop trials were created by combining color names (“RED,” “BLUE” and “YELLOW”) with ink colors (RGB color codes 255,0,0, 0,0,255 and 255,255,0, respectively). Three trials were congruent (e.g., “RED” written in red) and six were incongruent (e.g., “RED” written in yellow).

The Hot Stroop task displays black and white images of emotional faces (happy, angry and fearful) taken from the NimStim set of facial expressions [43] (two males and one female referred as the 24M, the 28M and the 10F in the NimStim set), and words naming the corresponding emotion (“HAPPINESS,” “ANGER” and “FEAR”), printed in 24-pt Courier New font capital letters. Stimuli sustained on average 4.12° x 10.05° of visual angle. For incongruent items, the emotional faces and the words were discordant (e.g., a happy face with the word “ANGER” beneath). Participants had to inhibit the meaning of the word (e.g., “ANGER”) to correctly identify the emotion displayed on the face (e.g., happy face). For congruent items, the emotional faces and the words were consistent with one another (e.g., a happy face with the word “HAPPINESS” beneath). Nine Stroop items were created by combining different emotional faces (happy, angry or fearful) with the three corresponding emotional words (“HAPPINESS,” “ANGER” and “FEAR”). Three items were congruent (e.g., a happy face with the word “HAPPINESS” beneath), and six were incongruent (e.g., a happy face with the word “ANGER” beneath).

#### Procedure

Participants were recruited by groups of three same age individuals coming from the same classroom for both adolescents and young adults. They were recruited from the same classroom at the middle schools or at the university and then tested in two other rooms within the schools or at the university. These groups were then randomly assigned to a Social evaluation condition (two individuals) or to an alone condition (one individual). Note that we did not control that participants know one another but they were in the same classroom for at least one course during the semester.

Under the alone condition, the participants were alone in the experiment room while performing the tasks. Each participant in the alone condition performed both the Hot and the Cool Stroop tasks. The task order of presentation was counterbalanced across participants. All instructions were provided in written and oral forms via the computer.

Under the social-evaluation condition, one member of the group was randomly chosen as the primary participant and the other as the evaluator. Thus, the peer was a same age classmate of the primary participant (i.e; same age adolescent in the adolescent group and same age young adult for the young adult group). Although most of the study matched the gender of the peer evaluator and the participant (see for example [25]), in the present experiment, the gender of the peer evaluator was matched with the participant’s gender for 56% of the participants and was different for 44% of the participant to control for gender effect. Importantly, the proportion did not differ between the age groups (χ^2^ = 0.9, p = .34).The experimenter informed participants that his/her classmate would be present in the room and would observe them to evaluate their performance using an evaluation form. The peer evaluator was trained to use the evaluation form in a separate room before the start of the experiment. Trained peers were instructed to evaluate the participant’s accuracy in filling out the evaluation form without interacting with the participant throughout the experiment. Specifically, they were instructed to estimate the number of errors made by the participants to unsure that they were actively engaged in social evaluation. Each participant was seated in front of the computer, and the evaluator was seated 70 to 80 cm to the right of the participant in such a way that the evaluator could observe both the computer screen and the participant’s responses. Each participant in the social evaluation condition performed both the Hot and the Cool Stroop tasks. The task order of presentation was counterbalanced across participants. All instructions were provided in written and oral forms via the computer. In the *Cool Stroop* task and the *Hot Stroop* task, participants were instructed to provide their responses by pressing one of three keyboard buttons associated with the three possible ink colors (i.e., “red,” “blue” and “yellow”) or the three possible emotions (i.e., happy, angry or fearful). Each stimulus remained on the screen until the participant responded, with a time limit of 3000 ms. Interstimulus intervals lasted 1000 ms and comprised a central black fixation cross. To maximize reactive inhibitory control in the *Cool Stroop* task and the *Hot Stroop* task, 75% of the trials were congruent, and 25% were incongruent. Thus, 54 congruent and 18 incongruent stimuli were presented randomly in each Stroop task.

As a manipulation check, at the end of the task, each participant completed a questionnaire to evaluate their feeling of embarrassment created by the evaluator (in the social context condition) or the experimenter (in the alone condition). Feeling of embarrassment was self-evaluated on a 7-point scale ranging from 1 (not embarrassed at all) to 7 (extremely embarrassed). The questionnaire was presented to the participants as a classical debrief of the experiment. Critically, ANOVA on the self-report embarrassment scores revealed a main effect of the condition, F(1, 97) = 4.01, p = .048, η_p_^2^ = .04, confirming that participants were more embarrassed in the Social evaluation condition than in the Alone condition. However, we found no main effect of age group, F(1, 97) < 1, nor interaction between age group and the social conditions, F(1, 97) = 1.08, p = .30, η_p_^2^ = .011.

### Results

Consistent with prior studies using the Cool and the Hot Stroop tasks [18], an interference score was computed on the response times (RTs) (i.e., difference in RTs between the incongruent and congruent items) and on the error rates (ERs). Note that lower interference scores reflect better inhibitory control performance (see Table 2). Only correct trials were included in RTs analyses. Trials exceeding two standard deviations (SD) from the participant’s mean for each of the condition of each Stroop were excluded from the analysis (2.5% in total). For each of the analyses, we provided an estimate of the effect size, the partial eta squared (*η*^*2*^_*p*_) for the analyses of variance (ANOVA) and Cohen’s d (*d*) for the planned comparisons.

**Table 2:** Mean RTs (ms), mean ERs (%) for the Cool and Hot Stroop items for each age group and each social context condition (Alone condition: AC; Social evaluation condition: SE; standard deviations are in parentheses and * indicated significant d ifference between the AC and the SC conditions).

				Cool Stroop task						Hot Stroop task					
				Congruent Items		Incongruent Items		Interference score RTs	Interference score Ers	Congruent Items		Incongruent Items		Interference score RTs	Interference score Ers
			N	RTs	ERs	RTs	ERs			RTs	ERs	RTs	ERs		
**Experiment 1**	**AC**	**Adolescents**	24	686.39 (131)	2.24 (3)	862.72 (246)	11.57 (12)	176.33 (152)	9.34 (11)	761.71 (182)	2.55 (3)	939.58 (284)	13.19 (12)	177.88* (142)	10.65 (14)
		**Young Adults**	24	576.80 (85)	1.85 (3)	697.40 (142)	4.63 (6)	120.60 (101)	2.78 (7)	657.92 (162)	2.55 (3)	715.34 (189)	5.79 (7)	57.42 (54)	3.24 (6)
	**SC**	**Adolescents**	26	633.11 (89)	2.35 (2)	771.74 (159)	8.55 (10)	138.63 (116)	6.20 (9)	748.81 (181)	3.06 (4)	852.40 (182)	14.32 (10)	103.60* (139)	11.25 9)
		**Young Adults**	27	568.29 (85)	4.13 (3)	690.92 (161)	4.12 (3)	122.63 (109)	0.01 (5)	606.62 (105)	2.13 (2)	680.81 (147)	5.76 (7)	74.19 (74)	3.64 (7)
**Experiment 2**	**AC**	**Adolescents**	21	686.66 (123)	2.03 (3)	893.86 (256)	12.96 (13)	207.20 (180)	10.93 (12)	763.62 (116)	3.62 (3)	969.47 (172)	15.34 (13)	205.86* (136)	11.73 (11)
		**Young Adults**	23	541.23 (72)	2.42 (4)	680.03 (150)	8.45 (9)	138.84 (109)	6.04 (8)	578.66 (69)	3.70 (4)	602.83 (93)	6.52 (7)	24.17 (68)	2.82 (7)
	**SC**	**Adolescents**	23	673.02 (116)	2.70 (2)	826.14 (232)	10.42 (10)	153.13 (156)	7.72 (10)	734.20 (144)	4.17 (4)	855.36 (197)	10.88 (10)	121.16* (109)	6.71 (9)
		**Young Adults**	22	585.18 (64)	3.37 (4)	710.90 (132)	11.62 (9)	125.72 (99)	8.25 (9)	609.23 (58)	2.86 (3)	643.44 (106)	10.10 (11)	34.21 (88)	7.24 (11)

Regarding the ERs interference scores, a 2 (Age group: adolescents vs. young adults) x 2 (social context: alone vs. social evaluation condition) x 2 (Stroop task: Hot vs. Cool) mixed-design ANOVA revealed a significant effect of the Stroop task, F(1, 97) = 4.95, p = .02, *η*_*p*_^*2*^ = .05, showing that the proportion of errors was higher for the Hot Stroop than for the cool Stroop. However there was no three-way interaction on the ERs interference scores, *F*(1, 97) < 1.

Regarding the RTs interference scores, a 2 (Age group: adolescents vs. young adults) x 2 (social context: alone vs. social evaluation condition) x 2 (Stroop task: Hot vs. Cool) mixed-design ANOVA revealed a main effect of the Stroop task, F(1,97) = 5.42, p = .02, η_p_^2^ = .05, showing that RTs interference scores were higher for the Cool Stroop than for the Hot Stroop. However, there was no three-way interaction on the interference scores, *F*(1, 97) < 1. Given that some of our hypotheses predicted specific effect of the social evaluation on the Hot Stroop task, the RTs interference scores were analyzed separately for each task. In addition, we provided either Bayes factors in favor of the null (*BF*_*01*_) or the alternative (*BF*_*10*_) hypothesis.

#### Cool Stroop task

The two-way ANOVA on the interference scores revealed no effect of age group, *F*(1, 97) = 2.23, *p* = .18, no effect of social context, *F*(1, 97) < 1, and no two-way interaction between age group and social context, *F*(1, 97) < 1, *BF*_*01*_ = 11.4 (strong evidence in favor of the null hypothesis).

#### Hot Stroop task

The two-way ANOVA on the interference scores revealed a significant main effect of age group, *F*(1, 97) = 11.84, *p* < .001, *η*_*p*_^*2*^ = .11 but no significant main effect of social context, *F*(1, 97) = 1.74, *p* = .19. Lastly, the two-way interaction between age group and social context was significant, *F*(1, 97) = 4.37, *p* = .04, *η*_*p*_^*2*^ = .04, *BF*_*10*_ = 7.8 (strong evidence in favor of the alternative hypothesis, “S1 Fig”).

Planned comparisons revealed that interference scores were lower in the social evaluation than under the alone condition for adolescents, *F*(1, 97) = 5.77, *p* = .02, *d* = .53, but not for young adults, *F*(1, 97) < 1.

### Discussion

Experiment 1 examined whether social evaluation by a peer influences inhibitory control performance similarly in cool and Hot contexts in adolescents and in young adults. To this end, adolescents and young adults performed a Cool and Hot version of the Stroop task, either alone or under the supervision of a peer evaluator.

The findings revealed that social evaluation by a peer facilitated adolescents’ performance in the Hot but not in the Cool Stroop task. In contrast, young adults showed no effect of social evaluation in either the Hot or Cool Stroop task. Taken together, Experiment 1 provides the first evidence that social evaluation by a peer can increase Hot inhibitory control performance specifically in adolescents. This result is consistent in part with the assumption that social context might influence inhibitory control in adolescents only under conditions of heightened affective arousal [28]. This result suggests that the salience of a peer to adolescents might be greater than to young adults. Our findings are in line with previous studies showing that the presence of an adult may facilitate the downregulation of risky behaviors in adolescents by increasing their inhibitory control performance [11, 14].

In contrast to studies in the field of social psychology that report a facilitation of cool inhibitory control under social evaluation in adults, the present results failed to reveal such effects for young adults or adolescents [9, 10]. This lack of effect of the social evaluation might be due to the nature of the social context induced in the present experiment. Indeed, previous studies have suggested that the social facilitation of cool inhibitory control performance appears only under the social evaluation of an expert adult [44, 45]. Consequently, the aim of Experiment 2 was to determine whether a social evaluation by an expert adult facilitates Hot and/or cool inhibitory control performance similarly in adolescents and young adults.

## Experiment 2 (expert adult evaluator)

To determine whether social evaluation by an expert adult influences the Hot and cool inhibitory control performance of adolescents and young adults, two new groups of 12-to-15-year-old adolescents and 18-to-23-year-old young adults performed the Hot and Cool Stroop tasks of Experiment 1 under either a social evaluation (i.e., in the presence of an expert adult evaluator) or an alone condition (i.e., tasks performed alone).

We reasoned that if the social facilitation of inhibitory control performance occurs only under social evaluation by an expert adult [30, 31], then performance in both Hot and Cool Stroop tasks should be higher in the social evaluation than under the alone condition in both adolescents and young adults. On the other hand, if social evaluation facilitates adolescents’ inhibitory control performance only under conditions of heightened affective arousal (see [28]), then consistent with the results of Experiment 1, adolescents should exhibit better performance in the Hot Stroop task under the social evaluation condition than under the alone condition.

### Material and method

#### Participants

Forty-five young adults and 45 adolescents were recruited in this experiment. None of them had participated in Experiment 1. The participants were randomly assigned to one of two social-context conditions: an alone condition with no social context, in which the participants were alone in the experiment room (alone condition, AC), and a social-evaluation condition, in which an adult was present to allegedly evaluate the participant (social-evaluation condition, SC). Table 1 displays the demographics of the four groups.

Sample size was determined a priori by running a priori power analysis using G*Power 3.1.9.2 [42], revealing that a minimum of 84 participants was required to detect a medium effect size of 0.25 (according to Cohen’s effect size conventions and based on previous studies reporting medium to large effect sizes of the social influence on risk taking and cognitive control, see for example [25, 37] on A 2 (Age group: adolescents vs. young adults) x 2 (social context: alone vs. social evaluation condition) x 2 (Stroop task: Hot vs. Cool) interaction with a power (1 - β) set at.80 and α set at.05.

We obtained informed written consent from parents and all adolescents. Participants were tested in accordance with national and international norms governing the use of human research participants. The Faculty of Psychology (University of Paris) granted the ethical permission to conduct this study.

#### Procedure

The material and the procedure used in Experiment 2 were strictly identical to the ones used in Experiment 1 except that the evaluator was an expert adult. In the SC, the experimenter informed participants that an expert in psychology and neuroscience would be present in the room and would evaluate their performance using an evaluation form. Expert adults (Two males and two females; M = 30.5 years old, SD = 4.4) were instructed to evaluate the participant’s accuracy in filling out the evaluation form without interacting with the participant throughout the experiment. To control for gender effect, the gender of the Expert evaluator was matched with the participant’s gender for 58% of the participants and was different for 42% of the participant. Importantly, these proportions did not differ significantly across the age groups (χ^2^ = 0.3, p = .58). Each participant was seated in front of the computer, and the evaluator was seated 70 to 80 cm to the right of the participant in such a way that the evaluator could observe both the computer screen and the participant’s responses.

As a manipulation check, ANOVA on the self-report embarrassment scores revealed a significant main effect of the condition, F(1, 86) = 4.7, p = .03, η_p_^2^ = .051, confirming that participants were more embarrassed in the Social evaluation condition than in the alone condition. However, there were no main effect of age group, F(1, 86) < 1, nor interaction between age group and the social conditions, F(1, 86) < 1.

#### Results

As in Experiment 1, the interference scores were computed on both the RTs (i.e., difference in RTs between the incongruent and congruent items) and the ERs (i.e., difference in ERs between the incongruent and congruent items) separately for each of the two Stroop tasks (see Table 2). Only correct trials were included in the analyses. For each of the analyses, we provided an estimate of the effect size as the partial eta squared (*η*^*2*^_*p*_) for the analyses of variance (ANOVA) and as Cohen’s d (*d*) for the planned comparisons.

Regarding the ERs interference scores, a 2 (Age group: adolescents vs. young adults) x 2 (social context: alone vs. social evaluation condition) x 2 (Stroop task: Hot vs. Cool) mixed-design ANOVA revealed no significant effect of the Stroop task, F(1, 86) < 1, nor three-way interaction on the ERs interference scores, *F*(1, 86) < 1.

Regarding the RTs interference scores, a 2 (Age group: adolescents vs. young adults) x 2 (social context: alone vs. social evaluation condition) x 2 (Stroop task: Hot vs. Cool) mixed-design ANOVA revealed no three-way interaction on the interference scores, *F*(1, 86) < 1. Following our hypotheses, the interference scores were analyzed separately for each Stroop task. In addition, we provided either Bayes factors in favor of the null (*BF*_*01*_) or the alternative (*BF*_*10*_) hypothesis.

*Cool Stroop task*. A two-way ANOVA with age group (adolescents vs. young adults) and social context (alone vs. social-evaluation conditions) as between-subject factors on the interference scores revealed no significant effect of age group, *F*(1, 86) = 2.65, *p* = .11, nor of social context, *F*(1, 86) = 1.30, *p* = .26. Finally, the two-way interaction between age group and social context was not significant, *F*(1, 86) < 1, *BF*_*01*_ = 7.0 (strong evidence in favor of the null hypothesis).

*Hot Stroop task*. A similar two-way ANOVA on the interference scores of the Hot Stroop task revealed a significant main effect of age group, *F*(1, 86) = 38.27, *p* < .001, *η*_*p*_^*2*^ = .31, and a marginal effect of social context, *F*(1, 86) = 2.96, *p* = .09, *η*_*p*_^*2*^ = .03. Finally, the two-way interaction between age group and social context was significant, *F*(1, 86) = 4.76, *p* = .03, *η*_*p*_^*2*^ = .05, *BF*_*10*_ = 10^5^ (strong evidence in favor the alternative hypothesis). Planned comparisons revealed that interference scores were lower in the social evaluation than under the alone condition for adolescents, *F*(1, 86) = 7.59, *p* < .01, *d* = .68, but not for young adults, *F*(1, 86) < 1(see “S1 Fig”).

## General discussion

The purpose of the present study was to determine whether social evaluation by a peer (Experiment 1) or by an expert (Experiment 2) influences cool and Hot inhibitory control performance similarly in young adults and in adolescents. To this end, adolescents and young adults performed a *Cool* and a *Hot* version of the *Stroop* task, either alone or under the supervision of a peer examiner (Experiment 1) or an expert adult (Experiment 2). Three major findings emerged: 1) Social evaluation of a peer increases Hot inhibitory control only in adolescents (Experiment 1); 2) a similar social facilitation effect was observed when adolescents were under the social evaluation of an expert (Experiment 2); and 3) social evaluation had no significant effect on the Hot and cool inhibitory control performance of young adults.

Taken together, these results indicate that social evaluation positively influences Hot inhibitory control in adolescence. As such, our results extend previous neuroimaging findings suggesting that peer presence does not influence inhibitory control [27, 28]. Although our findings are in line with the assumption that cool inhibitory control is not modulated by social context, the results reported in the two experiments provide evidence that social evaluation (by an expert or a peer) can increase inhibitory control performance under conditions of heightened affective arousal (i.e., Hot Stroop task) specifically during adolescence, a developmental period of hypersensitivity to the social context. These results are in contrast with studies showing that peer presence impaired adolescents’ inhibitory control performance in the context of social cues and anticipation of rewards [35]. However, our findings demonstrated that Hot inhibitory control might be facilitated under social evaluation by a peer or an expert even in the absence of reward. In addition, the differences observed between the present study and previous developmental investigations might be a consequence of the nature of the social manipulation. While previous studies have examined the influence of social context without explicit evaluation (i.e. mere social presence) [27, 28, 35], the present study involved an explicit social evaluation focusing on the participants’ performance during the tasks. Even if some evidences in the field of social psychology suggested that the social facilitation effect on the Stroop task exists not only under social evaluation but also under mere social presence [7, 9], some studies have reported that social evaluation can be detrimental for cognitive control as observed in previous developmental studies [7]. This performance decrements under circumstances of social evaluation is characterized as “choking under pressure” in the literature. Nevertheless, it should be noted that the reasons why social evaluation lead to facilitation effect in some situations (as in the present study), whereas it decrease performance in others are still debating and required further investigations. The social facilitation effect on Hot inhibitory control reported in adolescents advocates in favor of the distraction/conflict theory [37], which assumes that the social evaluation creates a distraction resulting in a cognitive overload that in turn reduces attentional focus and then facilitates inhibitory control. In contrast to studies highlighting the key role of expertise in the social evaluation effect [44, 45], our results revealed no significant modulation of cool inhibitory control performance by social evaluation in young adults. Given that the three-way interactions among age, social context, and the Stroop tasks failed to reach significance in either experiment, we cannot ignore that the absence of a social facilitation effect reported in the Cool Stroop reflects a lack of statistical power.

The present study presents several limitations. First, no measures of emotional responses, either by self-reports or physiological recording, were collected. In the future, it will be imperative to collect such measures to identify the nature of the process involved in each task. In line with this limitation, one could question whether the social facilitation effect observed in the Hot Stroop task might be due to the fact that Hot EFs tasks are more difficult than cool EFs tasks. Although this alternative interpretation could account for the social facilitation effect reported in Experiment 1—where we found higher error rates in the Hot than in the Cool Stroop tasks, it is unlikely to account for the social facilitation effect reported in Experiment 2 in which error rates did not differ between the two tasks. Thus, task difficulty alone cannot fully explain the social facilitation effects reported in our study. In addition, using social stimuli might have an effect on participants’ motivation. Nevertheless, social stimuli was used for both the congruent and the incongruent conditions of the Hot Stroop task. Therefore, it is likely that using interference scores allows to control for the potential effect of social stimuli on motivation. Moreover, one could argue that the lack of significant results of the social context in young adults might be explained by the smaller age difference between the participants and the adult examiner, leading young adults to consider the evaluator as a peer. Nevertheless, this hypothesis seems unlikely given that 1) the adult examiner was not only older than the participants but also introduced as an expert in the domain in Experiment 2, and 2) even a non-expert peer increased the Hot inhibitory control performance of adolescents in Experiment 1, suggesting that peer and adult evaluation have a similar facilitation effect on inhibitory control. Further research is needed to determine whether the age difference between participant and evaluator might modulate the impact of social context on inhibitory control.

## Conclusions

In conclusion, the present study is the first to provide evidence that social evaluation by a peer (Experiment 1) or by an expert (Experiment 2) facilitates Hot inhibitory control performance, specifically among adolescents. As such, the present finding expands our understanding of the positive influence of socioemotional context on inhibitory control during adolescence and offers new directions to investigate social influences in other cognitive domains in which inhibitory control is known to play a crucial role, such as in risky decision making.

## Supporting information

S1 Fig The interference effects on the response times (RTs) in the Cool (A) and the Hot (B) Stroop tasks of Experiment 1 and the Cool (C) and the Hot (D) Stroop tasks of Experiment 2 for each age group under each social condition. The error bars represent the standard error of the mean.
(PDF)

S1 Data (XLSX)
