# Supplementary figures and images for: How does social evaluation influence Hot and Cool inhibitory control in adolescence?
Source: PLoS One. 2021 Sep 30;16(9):e0257753. doi: 10.1371/journal.pone.0257753 (PMC8483316; doi:10.1371/journal.pone.0257753)

## Experiment 1

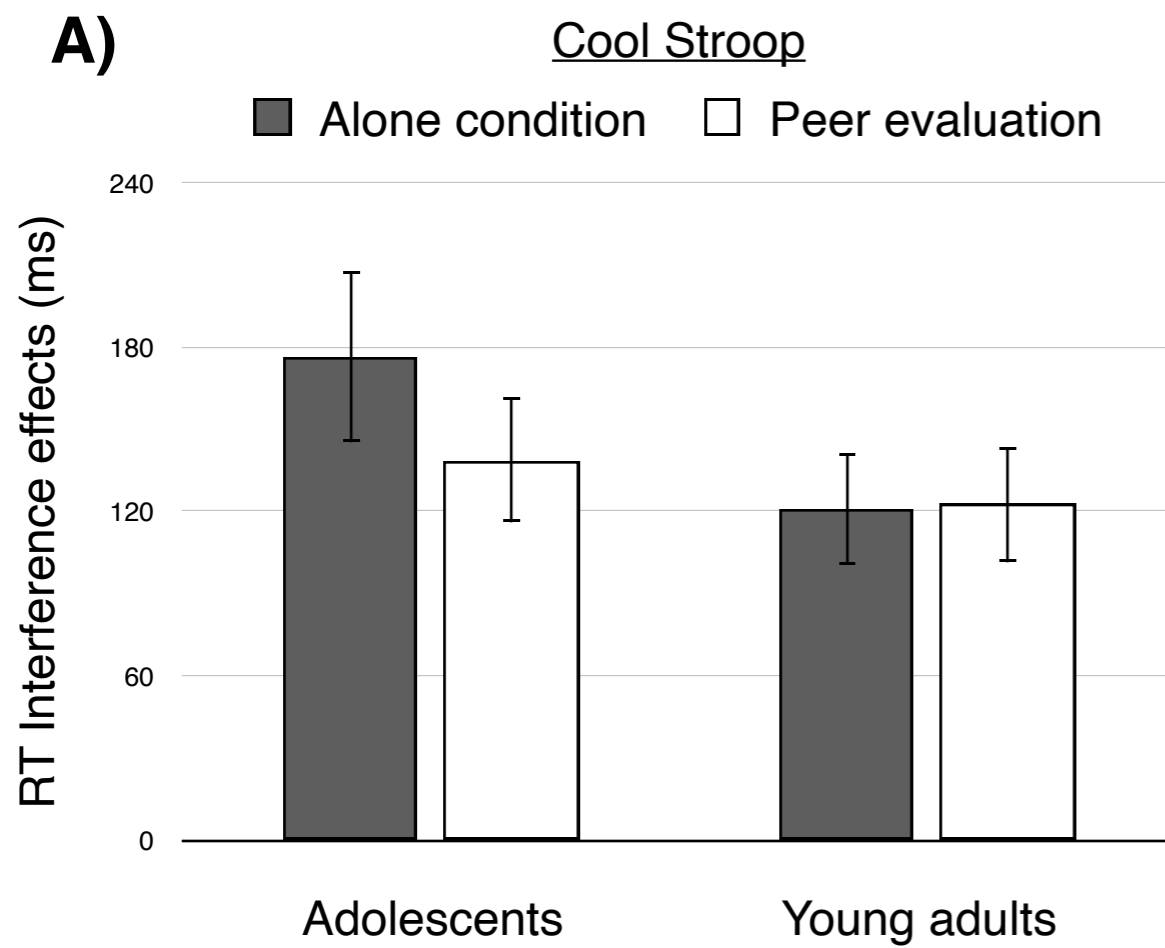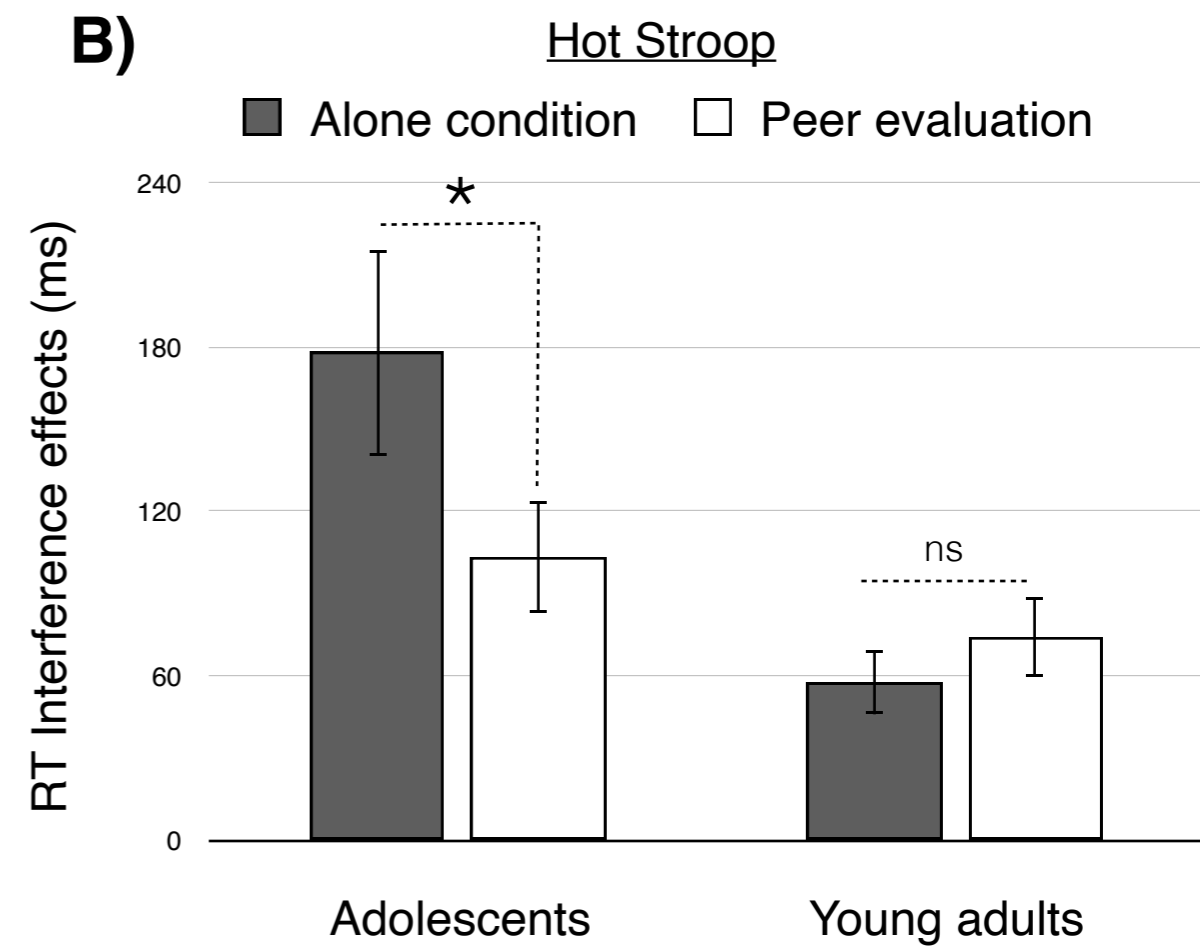

## Experiment 2

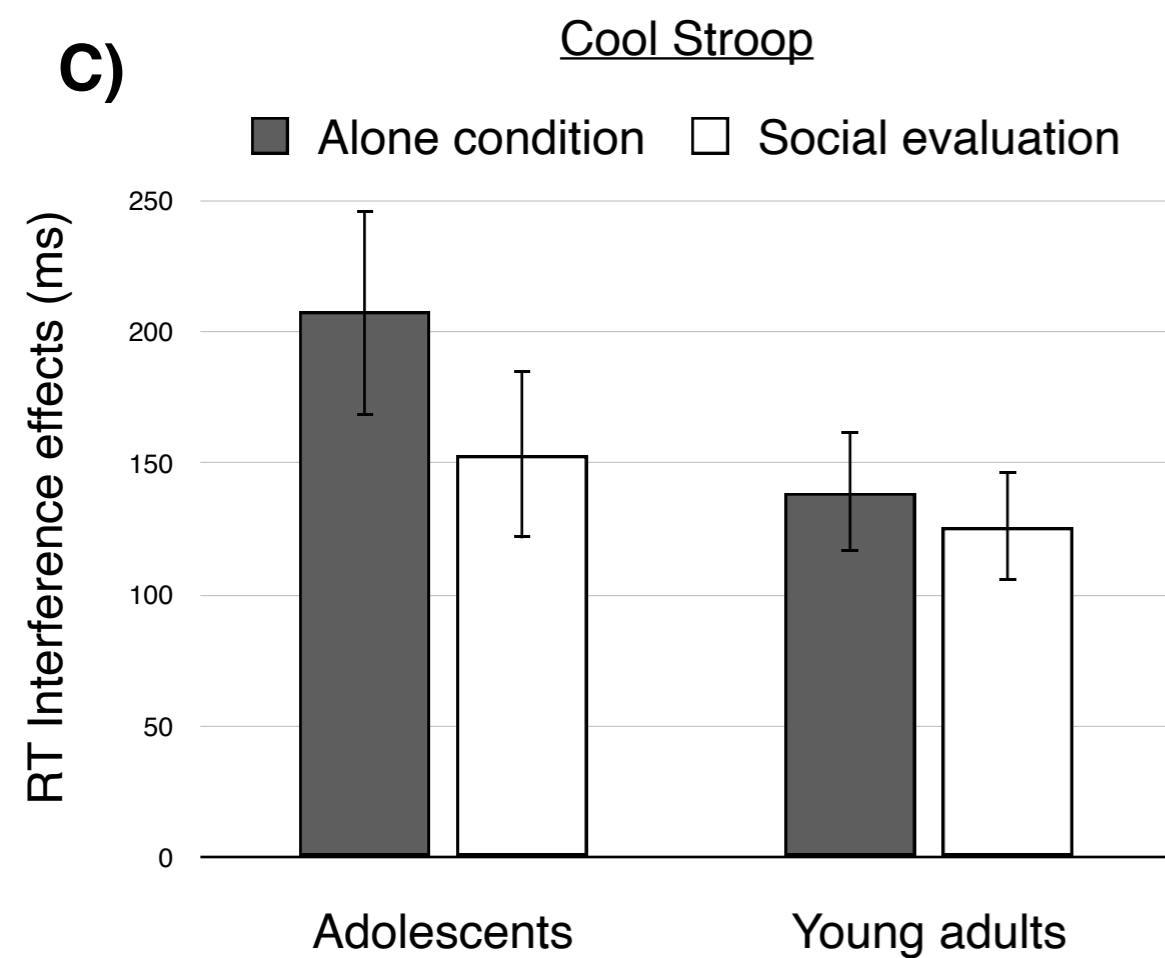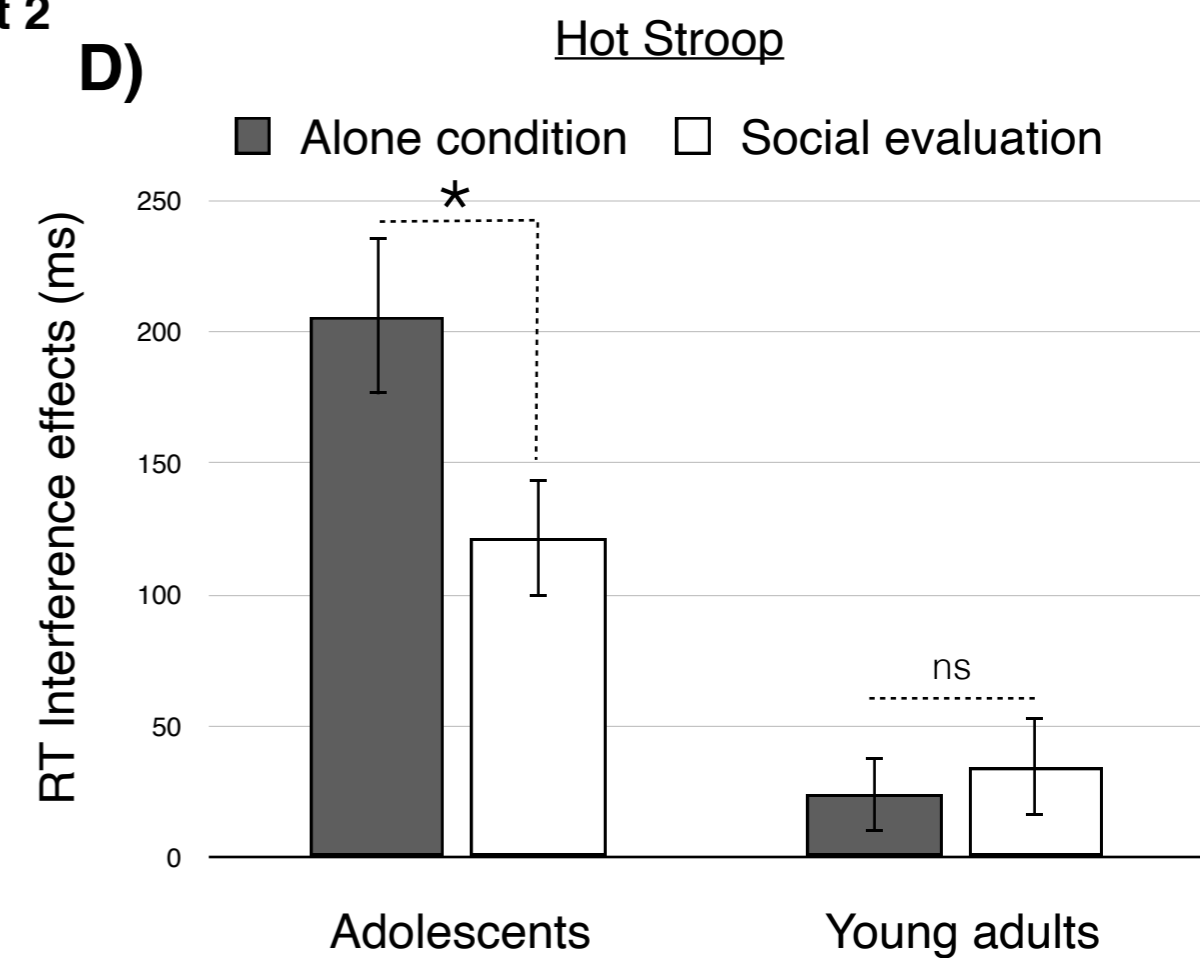

Supplement: S1 Fig — The error bars represent the standard error of the mean. (PDF) [file pone.0257753.s001.pdf]
